# Supplementary material for: Causal Relationship between Aging and Anorexia Nervosa: A White-Matter-Microstructure-Mediated Mendelian Randomization Analysis
Source: Biomedicines. 2024 Aug 16;12(8):1874. doi: 10.3390/biomedicines12081874 (PMC11351342; doi:10.3390/biomedicines12081874)
Supplement: Supplementary file 1 [file biomedicines-12-01874-s001.zip › Supplementary Figure/Figure S4/Singe SNP plot of MR analysis for TL to ubm-b-1836.pdf]

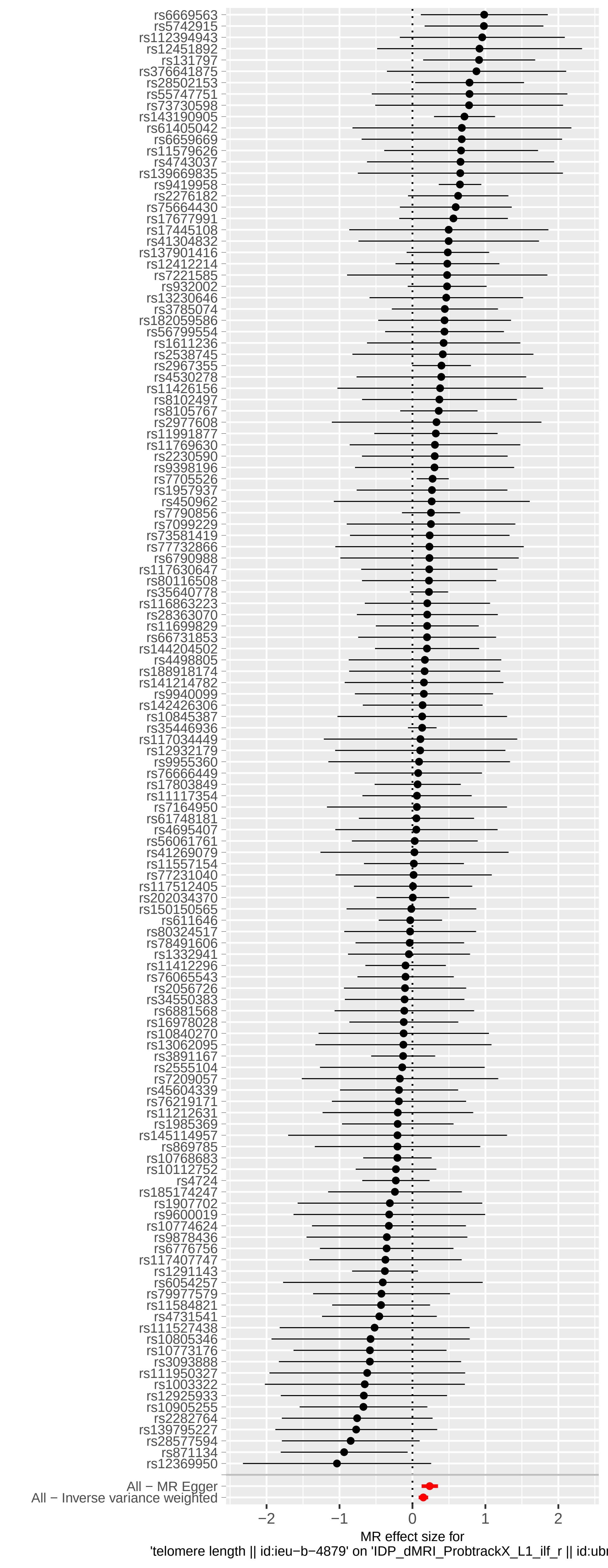
rs6669563
rs5742915
rs112394943
rs12451892
rs131797
rs376641875
rs28502153
rs55747751
rs73730598
rs143190905
rs61405042
rs6659669
rs11579626
rs4743037
rs139669835
rs9419958
rs2276182
rs75664430
rs17677991
rs17445108
rs41304832
rs137901416
rs12412214
rs7221585
rs932002
rs13230646
rs3785074
rs182059586
rs56799554
rs1611236
rs2538745
rs2967355
rs4530278
rs11426156
rs8102497
rs8105767
rs2977608
rs11991877
rs11769630
rs2230590
rs9398196
rs7705526
rs1957937
rs450962
rs7790856
rs7099229
rs73581419
rs77732866
rs6790988
rs117630647
rs80116508
rs35640778
rs116863223
rs28363070
rs11699829
rs66731853
rs144204502
rs4498805
rs188918174
rs141214782
rs9940099
rs142426306
rs10845387
rs35446936
rs117034449
rs12932179
rs9955360
rs76666449
rs17803849
rs11117354
rs7164950
rs61748181
rs4695407
rs56061761
rs41269079
rs11557154
rs77231040
rs117512405
rs202034370
rs150150565
rs611646
rs80324517
rs78491606
rs1332941
rs11412296
rs76065543
rs2056726
rs34550383
rs6881568
rs16978028
rs10840270
rs13062095
rs3891167
rs2555104
rs7209057
rs45604339
rs76219171
rs11212631
rs1985369
rs145114957
rs869785
rs10768683
rs10112752
rs4724
rs185174247
rs1907702
rs9600019
rs10774624
rs9878436
rs6776756
rs117407747
rs1291143
rs6054257
rs79977579
rs11584821
rs4731541
rs111527438
rs10805346
rs10773176
rs3093888
rs111950327
rs1003322
rs12925933
rs10905255
rs2282764
rs139795227
rs28577594
rs871134
rs12369950
All − MR Egger
All − Inverse variance weighted
−2
−1
0
1
2
MR effect size for
'telomere length || id:ieu−b−4879' on 'IDP_dMRI_ProbtrackX_L1_ilf_r || id:ubi
